# Protocol for evaluating physiological and psychological acclimatization mechanisms in Tibetan plateau environment: a clinical study of doctors from Peking Union Medical College Hospital

**DOI:** 10.3389/fpubh.2024.1490647

**Published:** 2024-12-24

**Authors:** Hemiao Xu, Daiyu Yang, Shuai Li, Kun He, Macuo Bian, Zhijuan Liu, Chengli Xu, Dong Wu

**Affiliations:** ^1^Chinese Academy of Medical Sciences and Peking Union Medical College, Beijing, China; ^2^State Key Laboratory of Complex Severe and Rare Diseases, Department of Gastroenterology, Peking Union Medical College Hospital, Chinese Academy of Medical Sciences and Peking Union Medical College, Beijing, China; ^3^Department of High Altitude Medicine, The People’s Hospital of Tibetan Autonomous Region, Lhasa, China; ^4^Department of Clinical Laboratory, The People’s Hospital of Tibetan Autonomous Region, Lhasa, China; ^5^United Laboratory of Polar Medical Sciences, State Key Laboratory of Medical Molecular Biology, Institute of Basic Medical Sciences, Chinese Academy of Medical Sciences, Beijing, China; ^6^Department of Gastroenterology, The People’s Hospital of Tibetan Autonomous Region, Lhasa, China

**Keywords:** physiology, psychology, high altitude, acclimatization, multiomics

## Abstract

**Introduction:**

The transition from low to high altitude environments is associated with a multifaceted series of physiological and psychological alterations that manifest over time. These changes are intricately intertwined, with physiological acclimatization primarily mediated through the regulation of hypoxia-inducible factor (HIF), which orchestrates the expression of critical molecules and hormones. This process extends to encompass the epigenome, metabolism, and other regulatory mechanisms. In the realm of psychological acclimatization, chronic hypoxia and changes in atmospheric pressure at high altitudes may contribute to decreased levels of neurotransmitters, with potential implications for mental health, particularly in relation to sleep quality. Despite significant advancements in our understanding of plateau acclimatization mechanisms in recent years, there remain many uncertain factors that necessitate further research.

**Methods:**

This study is a single-center prospective observational study. It aims to utilize a series of physiological and medical instruments in conjunction with internationally recognized physiological and psychological questionnaires to monitor the dynamic shifts in the acclimatization ability of doctors from Peking Union Medical College Hospital. The monitoring will occur at seven distinct time points: pre-departure from Beijing, 1–7 days post-arrival at the Tibetan plateau during the acute phase of plateau hypoxic stress, and during the chronic phase of plateau hypoxic stress at 2 weeks, 3 months, 6 months, 12 months of residency in Tibet, and post-return to Beijing. Concurrently, a spectrum of omics analyses will be conducted, including comprehensive genomic, proteomic, and metabolomic assessments of blood leukocytes, fecal, and oral samples.

## Graphical abstract

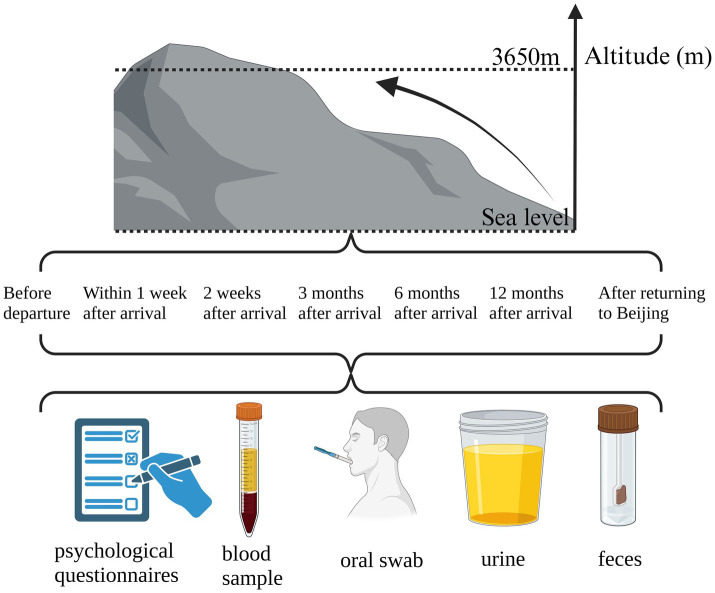

## 1 Introduction

More than 140 million people live at high altitude (above 2,500 m) (1). Among them, the major indigenous high-altitude populations live on the Andean, Tibetan, and east African plateau, where they experience the unique stress of hypobaric hypoxia (2).

Nowadays, more and more people are traveling to high altitude areas. The transition from low to high altitudes precipitates a multitude of physiological and psychological alterations in the human body, which may cause acute mountain sickness, high-altitude cerebral edema, and high-altitude pulmonary edema and so on (3). However, it has been established that mammals have evolved pivotal adaptive mechanisms to mitigate hypoxia, including enhanced ventilation, cardiac output, vascular proliferation, and the augmentation of circulating red blood cells. At the cellular level, ATP depletion responses are inhibited, and metabolic pathways are modulated to reestablish oxygen homeostasis (4). Furthermore, human responses to acute hypoxia include the activation of the adrenergic system and tachycardia, while hypoxic pulmonary vasoconstriction elevates pulmonary artery pressures. Over time, autonomic nervous system adaptations lead to a reduction in tachycardia, safeguarding the myocardium from excessive energy expenditure. Long-term exposure to high altitude results in cardiac adaptations with no obvious dysfunction (5).

The molecular underpinnings of altitude acclimatization have garnered considerable attention, with current research indicating that hypoxia-inducible factors (HIFs) play a pivotal role in orchestrating the expression of messengers and hormones, such as erythropoietin, vascular endothelial growth factor, and glucose transporters, in response to hypoxia (6). Moreover, the response to hypoxia encompasses a plethora of molecular and cellular alterations, including epigenetic modifications, non-coding RNA expression, metabolic shifts, signaling pathways, and various homeostatic mechanisms to ensure cellular survival under hypoxic stress (7).

Psychological alterations also accompany the physiological responses to hypobaric hypoxia and atmospheric pressure changes at high altitudes. Studies have indicated a correlation between increasing altitude and the prevalence of depression, attributing this to the challenging environmental and living conditions prevalent in high-altitude regions (8). Hypoxia and atmospheric pressure fluctuations may diminish serotonin levels in the brain (9), thereby compromising emotional regulation, potentially elevating the risk of depression (10). Additionally, sleep quality degradation is a common physiological effect at high altitudes, posing a significant threat to the physical and mental well-being of individuals residing or traversing these regions (11).

Despite the significant strides made in unraveling the molecular mechanisms of altitude acclimatization, the determinants of successful hypoxic acclimatization and the etiology and risk factors of altitude-related diseases remain elusive (12). Furthermore, research on psychological acclimatization to high-altitude environments is limited, warranting further exploration. Therefore, this study endeavors to elucidate the regulatory pathways that underpin the physio-psychological acclimatization of human beings in the Tibetan plateau hypoxic environment, with the aim of informing the development of optimized or novel preventive strategies for individuals ascending to high altitudes. The findings are anticipated to enhance the understanding of altitude sickness and potentially refine its treatment strategies.

## 2 Method

### 2.1 Design

This study is a single-center prospective observational study. It is intended to employ a suite of physiological and medical instruments in conjunction with internationally recognized standardized physiological and psychological questionnaires to dynamically monitor the physiological and psychological acclimatization of medical professionals from Peking Union Medical College Hospital. The monitoring will be conducted at seven key time points: prior to departure from Beijing, within 1–7 days post-arrival in Tibet during the acute phase of plateau hypoxia stress, during 2-week, 3-month, 6-month, and 12-month stays in Tibet during the chronic phase of plateau hypoxia stress, and upon return to Beijing.

The physiological parameters to be assessed include blood pressure, heart rate, fingertip venous oxygen saturation, hemoglobin concentration, hematocrit, physical phenotype, electrocardiogram conduction, cardiovascular function, and pulmonary function. Concurrently, questionnaires will be administered to assess acute mountain sickness, sleep quality, and emotional state. Blood, fecal, oral, and urine samples will be collected for further analysis, encompassing blood biochemical measurements, blood leukocyte whole gene expression profiling, blood proteomics, blood metabolomics, fecal metagenomics, fecal metabolomics, oral microbiota metagenomics high-throughput sequencing, and urine microprotein detection. Additionally, fundus examination and a six-minute walk test will be conducted.

**Figure 1:**
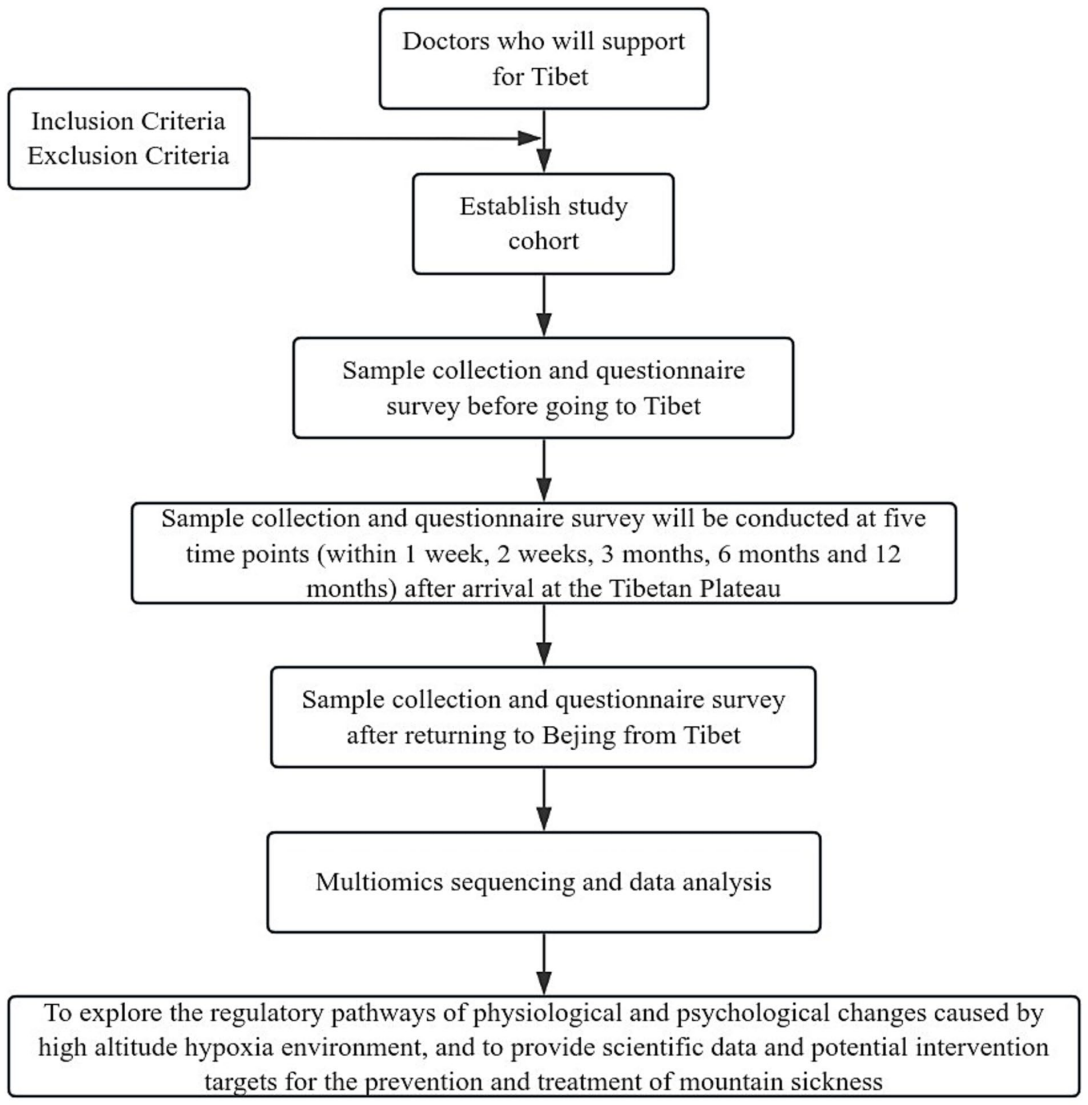
Flowchart of the study process.

In the present study, we aim to integrate the multi-omics sequencing outcomes obtained at seven distinct temporal intervals with the fluctuations observed in a array of biomarkers at corresponding time points. The objective is to delineate small molecules that are intrinsically linked to high-altitude acclimatization and to investigate the underlying molecular pathways. Concurrently, we propose to conduct a comparative analysis of omics sequencing data from groups stratified based on varying detection indicators across different time points. This approach is intended to elucidate the molecular mechanisms that may underpin the disparities in detection indicators. It is anticipated that these analytical endeavors will contribute to a more nuanced comprehension of altitude-induced illness and the physiological processes associated with altitude acclimatization. The trial design is presented in Figure 1, while the subsequent sections provide a comprehensive and detailed description of our trial.

### 2.2 Study population

The study will recruit 17 eligible medical professionals from Peking Union Medical College Hospital who are qualified to support Tibet and meet the basic requirements for physical examination, with a gender distribution of 10 males and 7 females. Participants must be between the ages of 20 and 65 years, able to provide written informed consent, and willing to cooperate. The inclusion and exclusion criteria will be strictly adhered to, ensuring the integrity and relevance of the sample.

#### 2.2.1 Inclusion criteria

(1) Healthy individuals aged 20–65 years.
(2) They can sign a written informed consent and is willing to cooperate.
(3) They have the qualification to aid Tibet and meet the basic requirements of physical examination for aid to Tibet.
(4) There are no acute or chronic illnesses, such as asthma or pulmonary edema.

#### 2.2.2 Exclusion criteria

(1) Age less than 20 years or more than 65 years.
(2) Unable to sign a written informed consent or unwilling to cooperate.
(3) They do not have the qualification for supporting Tibet or do not meet the basic requirements of medical examination for supporting Tibet.
(4) There are acute or chronic illnesses, which will increase risk of injury from exposure to high altitude and hypoxia.

#### 2.2.3 Withdrawal criteria

(1) Subjective willingness of withdrawal from the study because of various considerations after enrolment.
(2) Patients cannot complete the experiment for any reason.

### 2.3 Sample collection

#### 2.3.1 Basic physiological parameters

With respect to physiological parameters, the blood pressure, heart rate, and fingertip arterial oxygen saturation of the medical personnel are monitored utilizing an electronic sphygmomanometer and a pulse oximeter, respectively. A body composition analyzer is employed to quantify body weight, body mass index (BMI), body age, visceral fat index, basal metabolic rate, and the percentage of fat and muscle in various body segments.

#### 2.3.2 Cardiopulmonary function and fundoscopic examination

Regarding cardiopulmonary function assessment, a 12-lead electrocardiogram (ECG) device is utilized to examine the ECG conduction attributes, encompassing resting ECG and heart rate variability analysis. A cardiac function monitor is employed to assess cardiovascular function, including metrics such as heart rate, blood pressure, cardiac pumping and systolic functions, left heart workload, and peripheral vascular resistance. Pulmonary function testing is conducted to evaluate changes in maximum vital capacity, forced expiratory volume in one second (FEV1), and FEV1-to-forced vital capacity ratio (FEV1/FVC). Moreover, a 6-min walk test is administered to assess exercise endurance and cardiopulmonary function (13). Additionally, a fundoscopic examination is performed to inspect the vascular morphology of the fundus, to determine the presence of edema, and to ascertain any signs of hemorrhage (14).

#### 2.3.3 Subjective assessments

Subjective assessments are conducted using the Lake Louise Acute Mountain Sickness Symptom Rating Scale to gauge the incidence of acute mountain sickness among the medical support staff in Tibet (15). The Adult Self-rating Health Scale is employed to evaluate scores for anxiety, depression, hostility, and somatization symptoms (16). The Pittsburgh Sleep Quality Index is utilized to assess alterations in subjective sleep quality (17). The Depression-Anxiety-Stress Scale is used to gauge the emotional state of the Tibetan medical professionals (18). Additionally, a questionnaire survey is conducted to evaluate the perception of oxygenation, oxygen comfort, thermal sensation, and thermal comfort under various experimental conditions (19).

#### 2.3.4 Biological sample collection

For the procurement of blood, stool, urine, and oral samples, cubital vein blood is drawn for the analysis of hemoglobin concentration (20), hematocrit (HCT), and the calculation of the ratio of blood oxygen saturation to HCT. Further biochemical analyses included glucose (GLU), lipase (LIP), serum testosterone (T), ferritin (FERR), progesterone, vascular endothelial growth factor (VEGF), myoglobin (MYO), human interleukin-1β (IL-1β), total bilirubin (BILT), and alpha-amylase pancreatic (AMY-P) levels (21), as well as omics analyses. Fecal and oral samples are collected for subsequent metagenomic and fecal metabolomics high-throughput sequencing. Urine samples are analyzed for urinary α1-microglobulin (α1-MG), urinary β2-microglobulin (β2-MG), and microalbumin (mAlb) (22). Oral microbial community samples are obtained from participants prior to any eating, drinking, or tooth brushing upon waking in the morning. Sterile cotton swabs are used to thoroughly swab and rotate on the buccal mucosa, tongue base, and saliva, with the swab tips then placed in cryopreservation tubes and stored at −80°C pending metagenomic sequencing (23).

### 2.4 Omics data analysis

#### 2.4.1 Whole genome expression profiling of blood leukocytes

The acquired genomic data is subject to rigorous preliminary quality control measures, which entailed the elimination of low-quality reads and the excision of adapter sequences. Subsequent to these preprocessing steps, high-quality reads are aligned to a reference genome using established bioinformatics protocols, which may include tools such as STAR or Bowtie2. Gene expression levels are quantified through the application of bioinformatics tools such as HTSeq or Cufflinks. Differential expression analysis is conducted utilizing specialized statistical software, such as DESeq2 or EdgeR, to pinpoint genes exhibiting significant alterations in expression across various time points and environmental conditions, which may be implicated in the acclimatization to hypoxic stress.

#### 2.4.2 Proteomic analysis of blood samples

Following data acquisition, proteomic data is processed using sophisticated bioinformatics software, encompassing peptide identification, protein inference, and quantitative analysis. Initially, raw mass spectrometry data is analyzed to identify peptides. Algorithms such as Mascot, Sequest, or MaxQuant will be employed to enhance accuracy and sensitivity in identifying peptides with high confidence. Then comes the protein inference, where the identified peptides are mapped back to their corresponding proteins, using probabilistic models. After that, quantitative analysis is performed to measure the abundance of proteins in the samples. Techniques such as label-free quantification, tandem mass tags (TMT), or isobaric tags for relative and absolute quantitation (iTRAQ) may be utilized. This approach is employed to delineate alterations in the blood proteome, thereby enhancing our understanding of the biological responses to health, disease states, and environmental stimuli.

#### 2.4.3 Blood metabolomics analysis

The mass spectrometry-derived data is subjected to bioinformatics processing, including the identification and quantification of spectral peaks, normalization, and correlation with a comprehensive database of known metabolites. Advanced algorithms are utilized to detect and identify spectral peaks within the mass spectrometry data. Once the peaks are identified, quantification is performed to measure the concentration of each metabolite. To account for variations in sample preparation and instrument sensitivity, normalization techniques are applied. The identified metabolites are then correlated with a comprehensive database of known metabolites. After these processing steps, statistical analysis and data visualization techniques are employed to compare the metabolite profiles and concentrations under varying conditions, thereby elucidating the impact of hypoxia on physiological function.

#### 2.4.4 Fecal and oral metagenomic analyses

The raw sequencing data obtained is meticulously quality-controlled to exclude sequences of low quality and those not of microbial origin. Subsequent to this, sequence assembly, operational taxonomic unit (OTU) clustering, and species annotation are executed using advanced bioinformatics tools, such as QIIME or Mothur. A detailed examination of the microbial community structure and function is conducted through a series of analyses, including diversity assessments using metrics like Shannon and Simpson indices, species abundance statistics, and functional prediction, thereby providing insights into the correlation between gut microbiota and the health status of the host.

#### 2.4.5 Fecal metabolomics analysis

The spectroscopic data from fecal metabolomics undergoes thorough processing using advanced bioinformatics software. This begins with peak detection. Following peak detection, metabolites are identified and quantified by comparing detected peaks against established databases, such as the Human Metabolome Database (HMDB). Normalization techniques are applied to standardize the data, addressing variations from sample preparation and analytical conditions. By comparing metabolite signatures across different samples, this approach aims to identify metabolic shifts linked to hypoxic conditions. Such analysis provides insights into how the host’s metabolism adapts to low oxygen levels. Moreover, it highlights the interplay between the host and gut microbiome, as metabolites produced by microbial metabolism can significantly influence host physiology.

#### 2.4.6 Multi-omics analysis of blood, stool and oral samples

Finally, a comprehensive bioinformatics framework is established for integrating and analyzing multi-omics data from blood, fecal, and oral samples. This integrative approach combines genomic, proteomic, metabolomic, and metagenomic data to provide a holistic understanding of biological systems.

The process begins with data harmonization to standardize different omics datasets, ensuring compatibility for analysis. Advanced statistical methods and machine learning algorithms are then employed to identify cross-omics patterns and correlations. Techniques like principal component analysis (PCA) and clustering help reveal significant trends and relationships.

Network analysis further elucidates interactions between biological molecules, allowing researchers to visualize complex relationships among genes, proteins, and metabolites. This helps identify key hubs and pathways involved in the acclimatization of Tibetan individuals to the hypoxic plateau environment.

### 2.5 Statistical analysis of detection indicators

SPSS V.26.0 will be used as the statistical software. Quantitative data with a normal distribution will be presented as means with standard deviations and will be compared between groups with the use of Student’s t-test or the rank-sum test. Quantitative data that do not follow a normal distribution or homogeneity of variance will be presented as median and IQR, and comparisons between groups will be performed by the Mann–Whitney U test. Enumeration data will be presented as ratios and percentages, and χ^2^ tests will be used for comparison. Two-tailed *p* < 0.05 is considered statistically significant.

### 2.6 Trial management

The selection and sampling of study subjects will be carried out in strict accordance with the study design requirements. We will implement a document management approach to ensure data authenticity, completeness, reliability, and comparability. The analysis of the experimental data will be scientific, the research report will be authentic and rigorously written, and the research data will be properly archived and stored in accordance with the research storage guidelines.

### 2.7 Patients and public involvement

Patients and/or the public are not involved in the design, or conduct, or reporting, or dissemination plans of this trial.

### 2.8 Confidentiality

In the course of this investigation, personal and medical information pertaining to the study participants may be gathered or processed, encompassing, but not limited to, the following details: full name, gender, date of birth, residential address, contact telephone number, and diagnostic outcomes of the medical professionals providing support in Tibet. The personal data of the Tibet-supporting physicians will be utilized exclusively for the objectives delineated within the study’s protocol. Medical information obtained by the Tibet-supporting physicians affiliated with the institution will be maintained with strict confidentiality. Publication of study findings in academic periodicals will be conducted in a manner that precludes the disclosure of any personally identifiable information that could trace back to the physicians supporting Tibet. The principal investigator assumes responsibility for the secure storage and appropriate utilization of all personal data pertaining to the Tibet-supporting medical professionals involved in the study. The ethics committee or the clinical research regulatory authority may gain access to the personal data of these professionals as required. The research team will exert every feasible measure to safeguard the privacy of the personal medical data of the supporting physicians, in full compliance with legal provisions.

### 2.9 Data management

The sample collection mentioned in section 2.3 will be conducted by experienced doctors, and during the period in Tibet, the same group of doctors will complete the task to ensure the avoidance of potential biases. The subjective questionnaires will be distributed online to participants in the form of electronic questionnaires, which participants will complete on their own mobile phones. The collected biological samples will be stored in a dedicated biobank refrigerator at Peking Union Medical College Hospital, and the other information will be saved on an in-house computer at Peking Union Medical College Hospital, which is accessible only to specific members of this research team, fully ensuring the security and privacy of the data.

## 3 Strengths and limitations of this study

(1) The study is anticipated to enhance our comprehension of the physiological underpinnings and psychological manifestations of hypoxic acclimatization.
(2) By concurrently monitoring physiological and psychological indicators at the same time points, this study aims to elucidate the potential correlation between these two domains.
(3) The study is expected to contribute to the identification of etiological factors and risk profiles associated with altitude-related illnesses.
(4) The sample size is anticipated to consist of 17 volunteers, which may pose challenges to statistical robustness and generalizability.

## 4 Ethics and dissemination

The Peking Union Medical College Hospital’s Research Ethics Committee has provided ethical clearance for this study, designated with the approval code 2022-I2M-1-003. The study protocol has been sanctioned and registered with the ClinicalTrials.gov, bearing the registration number NCT06557928. The results of this trial will be disseminated in an open-access format, with the intention of sharing the findings among medical professionals.

## 5 Discussion

This study aims to conduct an exhaustive examination of the impact of high-altitude hypoxia acclimatization on human physiological and psychological metrics, as well as to investigate the potential interconnection between these two domains. The research endeavors to delineate the regulatory pathways underlying the physiological and psychological phenotypes elicited by hypoxia in the Tibetan plateau, thereby providing scientific evidence and identifying potential targets for the medical prophylaxis and treatment of altitude-induced illness. Furthermore, the findings are intended to furnish medical expertise for the development of work plans for personnel deployed to support medical services in Tibet.

The cohort of this investigation comprises medical practitioners from the Peking Union Medical College Hospital who are engaged in providing healthcare services in Tibet. The hospital’s robust management infrastructure facilitates the efficient procurement of data over the course of the two-year study period. Nonetheless, the limited sample size inherent to this study may present challenges in the interpretation of omics findings and the conduct of statistical analyses. Should circumstances permit, subsequent experimental iterations may augment the sample size to enhance the robustness and generalizability of the results.
